# Supplementary material for: Mask or no mask for COVID-19: A public health and market study
Source: PLoS One. 2020 Aug 14;15(8):e0237691. doi: 10.1371/journal.pone.0237691 (PMC7428176; doi:10.1371/journal.pone.0237691)
Supplement: Attachment — Submitted filename: Response to Reviewers.docx [file pone.0237691.s001.docx]

Dear Dr. Thavorn:

Thank you very much for this opportunity to revise and resubmit our manuscript. We now present our detailed responses to you and the reviewer’s comments, with the comments reproduced in italics followed by our response.

Comments from the editor:

1. *In the introduction and discussion, please include a new WHO recommendation on the use of masks in the community. How this revised recommendation may have impact on the effectiveness of a mask (Meff)?*

Responses: The WHO recommendation has been included in the revised manuscript, introduction page 3 line 58 to 59. “Our findings are consistent with the WHO’s advice on the use of masks in the context of COVID-19”.

2. *Please check the figure legends. Is there a typo in the description of Figure 2? Does Figure 1A refer to Figure 2A?*

Responses: The typo has been corrected. “Figure 1A” has been corrected as Figure 2A.

Comments from the reviewers

*Reviewer #1: The authors use a mathematical model to evaluate the impact of wearing masks on the burden of disease. Their model is well formulated, although rather simple. The results have good implications for public health policy and shows the effectiveness of wearing masks. I have some comments, and should these comments be addressed, I recommend the manuscript for publication.

1) Is it the case that the model uses a constant infectiousness rate throughout the natural course of infection? Does the level of infectivity change as the individual moves through their infection phase?*

Responses: In our current model, the level of infectivity changes when individuals are hospitalized (due to implied isolation). The default infectious rate is an average extracted from actual data (β _mild_)**.** It ranges from 0.6 to 0.1 in the model. The infectious rate is now explained in better detail in **Results and Discussion**, Transmission model (page 13, lines 260 to 263). We copy it here for your convenience.

“We also model that the infectivity level changes when the patient is admitted to the hospital (considered isolated). Based on earlier actual COVID-19 data fittings, it is estimated that the infection rate is in a range of 0.65-0.8 [71]. In our modeling, we set the rate to be 0.6 before hospital admission. We set the rate to be 0.1 after the individual is admitted.”

*2) Building on the comment above, it has been established presymptomatics have a substantial role in disease transmission. The viral load is highest immediately before symptom onset. Since the authors used a SEIR model in which the exposed compartment is not transmitting, this is a major limitation. If feasible, the model should be extended to contain a presymptomatic compartment as well. If this is not feasible, the limitation should be discussed.*

Responses: We appreciate the reviewer pointed out the pre-symptomatic patients could spread the virus. We have discussed the asymptomatic individuals’ role in our last submission (original submission pages 7-8 and 16). Particularly, in the section of “Model Rationale and Limitations” (original submission page 16), we direct future study to the impact of asymptomatic individuals on virus transmission. We copy it here for your convenience (original submission page 16 line 338-340, new version page 18 line 377-379).

“Further studies on asymptomatic individuals wearing a mask could be more convincing to validate the effectiveness of wearing a mask to prevent pre-symptom transmission.”

Moreover, the scientific community has not reached a consensus on the infectivity differences between “asymptomatic individual” and “pre-symptomatic individual” populations. In our original manuscript, we characterize that the “asymptomatics” individual could develop symptoms later, and thus falling into the “pre-symptomatic” category. We added more references for “pre-symptomatic”. The new edits in the revised manuscript read as (page 8 line 143-154):

***“*Asymptomatic, pre-symptomatic individuals and impacts on transmission**

There are documented cases remaining asymptomatic throughout the duration of laboratory and clinical monitoring [34, 36-39]. In many cases, a great portion developed some symptoms at a later stage thus are “pre-symptomatic” [40-45]. Studies also suggested that asymptomatic patients could spread the virus as their viral loads have no significant differences compared to those on the symptomatic patients [46, 47]. As such, pre-symptomatic transmission was estimated to have a shorter serial interval of COVID-19 (4.0 to 4.6 days) than the mean incubation period (five days) [12]. As a result, many secondary transmissions could have happened before the symptomatic cases are detected and isolated [48, 49]. Notably, Taipiwa et al. report the pre-symptomatic transmission composed 48% (95% CI 32-67%) for the Singapore outbreak and 62% (95% CI 50-76%) for the Tianjin outbreak in China [50].”

*3) The predicted numbers seem too high and not very realistic. However, this is expected given the homogenous nature and random mixing of the population. This should be discussed.*

Responses: We recognize the predicted numbers in the two scenarios “infection without intervention” and “intervention with relaxed social distance” are high compared to the reported infection cases at this time point. We also noted that on June 25^th^, 2020, CDC recognized the real infected cases could be 10 times more than the reported cases (US CDC news brief). However, to be consistent with the current available disease records and statistics, we added the discussion on page 13 and the new edits now read as follows (page 14 line 275-284):

“On June 14^th^, US has 2,081,296 confirmed cases when many states reopen, with cities and states recording increasing rates of new coronavirus cases per day. Since our model utilizes random mixing and assumes that all hosts have identical rates of disease-causing contacts with a homogenous nature, we expect individual behaviors and heterogeneity would profoundly impact the epidemic curve across the country. As a result, our model outputs could be higher than the real cases at a given time point. However, our purpose is to model the face mask impacts on the epidemic curve in a general population without considering other compounding factors. The SEIR model thus represents a good rational to evaluate face mask impacts.”

*4) There are a few typographical and grammar errors. For example, on line 121 the authors have "(COIVD, SRAS)".*

Responses: We appreciate the reviewer pointed out the typos and grammar errors. The typo has been corrected throughout the manuscript.

Other than the required edits from the reviewer and editor, we also added new references when we have a statement. We updated the references since some previously cited “under review” or “non-peer reviewed” articles are published. We have used ELSEVIER’s editorial service to edit and proofread the manuscript. The certificate of language editing use is attached. We hope you find this revised version meet the publication standards by PLOS ONE. We look forward to hearing from you.

Best regards,

Susie Y. Dai, PhD

sydai@exchange.tamu.edu

Associate Professor

Bioenvironmental Sciences Program

Plant Pathology and Microbiology

Texas A&M University

College Station, TX 77843

Office: 979-458-6362
